# Evaluation of heterotrophic plate and chromogenic agar colony counting in water quality laboratories

**DOI:** 10.1016/j.mex.2015.10.003

**Published:** 2015-10-22

**Authors:** Gary Hallas, Paul Monis

**Affiliations:** a Australian Water Quality Centre, SA Water Corporation, Adelaide, South Australia 5000, Australia; b School of Earth and Environmental Sciences, The University of Adelaide, Australia

**Keywords:** Digital, Heterotrophic plate count, Water, cfu, Membrane filtration, Chromogenic agar

## Abstract

The enumeration of bacteria using plate-based counts is a core technique used by food and water microbiology testing laboratories. However, manual counting of bacterial colonies is both time and labour intensive, can vary between operators and also requires manual entry of results into laboratory information management systems, which can be a source of data entry error. An alternative is to use automated digital colony counters, but there is a lack of peer-reviewed validation data to allow incorporation into standards. We compared the performance of digital counting technology (ProtoCOL3) against manual counting using criteria defined in internationally recognized standard methods. Digital colony counting provided a robust, standardized system suitable for adoption in a commercial testing environment. The digital technology has several advantages:

• Improved measurement of uncertainty by using a standard and consistent counting methodology with less operator error.
• Efficiency for labour and time (reduced cost).
• Elimination of manual entry of data onto LIMS.
• Faster result reporting to customers.

## Graphical abstract

**Figure:**
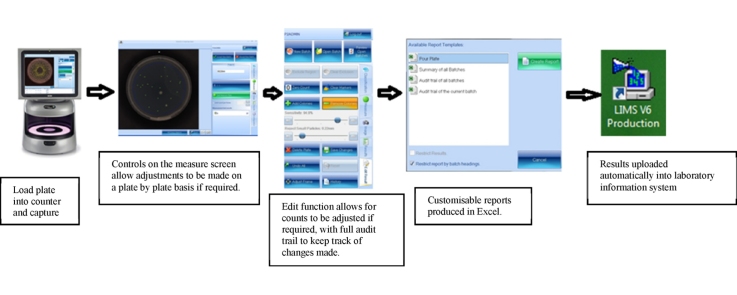

## Method details

The heterotrophic plate count (HPC) technique [1], [2] is well established for estimating the number of colony forming units (cfu) of aerobic and facultative anaerobic heterotrophic microorganisms present in a water sample. Similarly, membrane filtration (MF) using MI chromogenic agar has been shown to be an acceptable alternative to standard methods for the detection of faecal indicator bacteria [3], [4], [5], [6]. Manual colony counting introduces the potential for inter-operator variability, data entry errors and has a labour/time requirement that reduces efficiency and increases the cost of plate counting methods. There are a few studies reporting on the automated counting of bacterial colonies using digital imagining systems [7], [8], [9], [10], these studies demonstrated the potential application if this technology as a research tool but did not evaluate commercially available platforms and were not conducted with sufficient rigour to satisfy international standards for method validation.

We compare the use of digital counting technology (ProtoCOL3) against manual counting using criteria defined in internationally recognized standard methods [11], [12], [13], [14]. The ProtoCOL3 has LED lighting configured for high definition, with colour images taken using a 1.4 megapixel CCD camera. Resolution of the camera is 1 × 1 pixel equaling 42–43 μm. Colour classification is automatically self-correcting, using a density standard imaged with every plate, producing consistent colour detection within 1% against the “real-world” colour selection by the operator. Colours were chosen by selecting a representative number of colonies, individual pixel colour selection was from the software colour palate. Representative colonies are only limited by the number the operator chooses to select and categorize. Membrane filter grids were auto-detected and removed from the counting area by the software.

## Bacterial isolates

Suspensions were prepared using reference cultures or BioBalls (BTF, Precise Microbiology, Australia) as per the manufacturer's instructions for each reference culture requiring enumeration and colour discrimination by the digital counter. Each control (*Escherichia coli* – ATCC 11775, *Pseudomonas aeruginosa* – ATCC 10145) was performed at the beginning of each batch by all operators and the digital counter.

## Heterotrophic plate count

1. Yeast extract agar was prepared in accordance with the Water Microbiology Australian Standard “Heterotrophic colony count methods – pour plate method in 90 mm petri dishes” [1].
2. Quantities of the sample (1 mL) or dilutions of the sample were introduced into an appropriate petri dish and mixed with a tempered agar (48 °C), which contained non-limiting growth substrates for microorganisms.
3. Plates were incubated aerobically in a humid atmosphere at 35 ± 0.5 °C for 48 ± 3 h.

## Membrane filtration using chromogenic agar (MI agar)

1. MI agar chromogenic technology (BD Difco™, Becton Dickinson Diagnostics, USA) uses synthetic substrates for specific enzymatic reactions (such as β-glucosidase or β-glucuronidase activity) and relies primarily upon colour discrimination and enumeration [4].
2. Water samples from different matrix types (100 mL) were filtered through 0.45 μm sterile filters (Millipore, USA) according to Standard Methods for the Examination of Water and Wastewater method 9222B [15].
3. All MI agar plates were incubated for 24 h at 37 °C.

## Statistical analysis

All data were analyzed by criteria stipulated in ISO 17994:2004 – water quality, criteria for establishing equivalence between microbiological methods [14]. The recovery data in cfu per 1 mL for the heterotrophic plate count and cfu per 100 mL for chromogenic MI agar were tested for normality by using the Shapiro–Wilk test including the confidence interval (CI) expressing a range likely to contain the true value of a population statistic and the standard error (SE) measuring the variability from the mean.

The data for all media were tested for normality by using the Shapiro–Wilk test. The data sets that were not normally distributed were analyzed using non-parametric Kruskal–Wallis analysis of variance (KW-ANOVA) with the Dunn's post hoc test for significance, and Spearman-rank correlation coefficient (rs). Normally distributed data were analyzed using ANOVA with Tukey's post hoc analysis and Pearson's correlation coefficient (*r*).

The mean relative difference was used to compare the relative performance of the trial method versus the manual (reference) method. This was achieved by calculating the standard uncertainty (*U*) of counting, which is the relative standard deviation of results of repeated counting of the colonies from the same plate(s) under certain conditions. This is used to evaluate the comparison of the confidence interval of the expanded uncertainty around the mean. The trial method was considered “not different” (−10% ≤ *x*_L_ ≤ 0 and *x*_H_ > 0) for the trial methods with the lower and higher confidence intervals of the expanded uncertainty around the mean (*x*_L_ and *x*_H_) as the criteria is stated in ISO 17994:2004 Section 7.3 [14].

ISO/TR 13843 – water quality, guidance on validation of microbiological methods was used to determine other statistically relevant determinations of sensitivity and specificity, Section 9.2.

All statistical analyses were performed using Analyze-it for Microsoft Excel (http://www.analyse-it.com) and GraphPad Prism Version 4.03 for Windows (GraphPad Software, San Diego, CA, USA).

## Colony counter protocol flowchart

**Figure:**
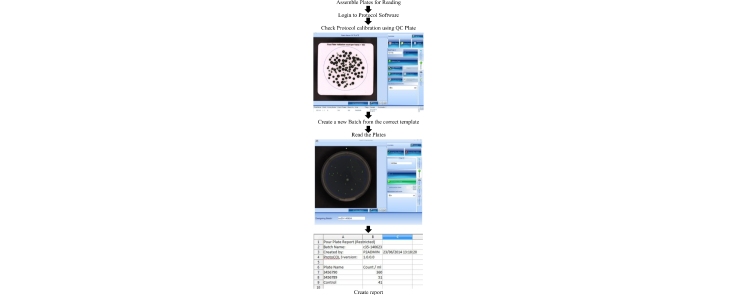

**Figure:**
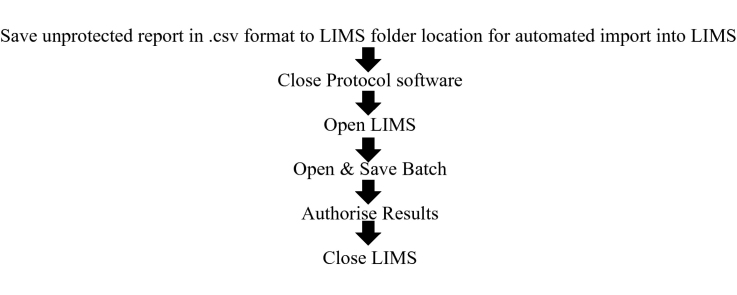

## Comparison of manual vs. digital counting

**Fig. 1:**
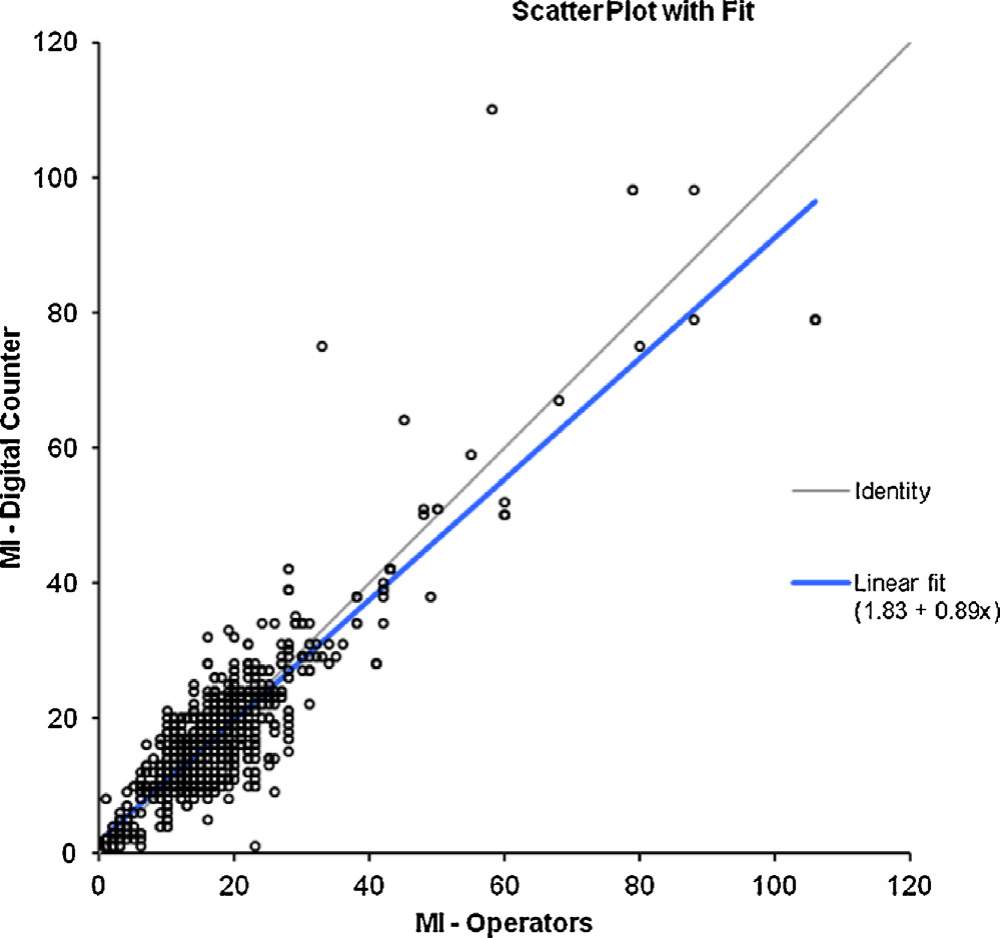
MI – method comparison of manual vs. digital method.

**Fig. 2:**
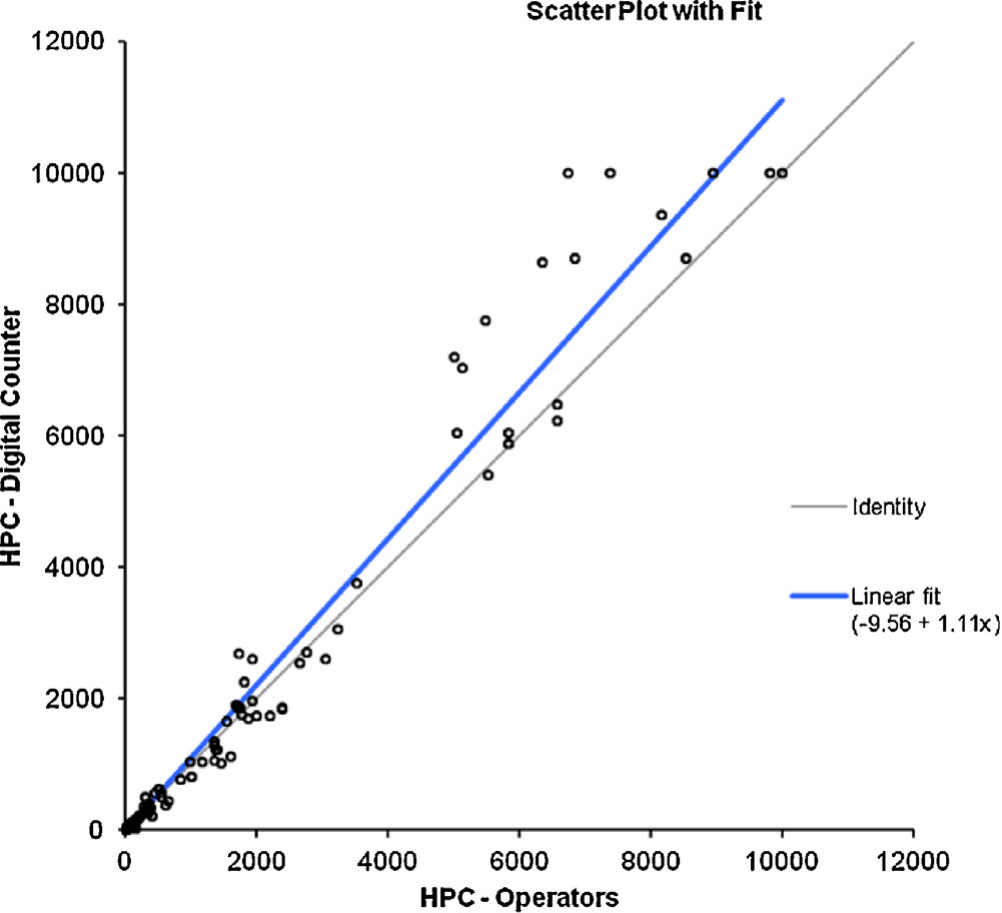
HPC – method comparison of manual vs. digital method.

The advent of digital colony counters (e.g. ProtoCOL3) removes the need for laborious manual counting and manual data entry for the enumeration of indicator organisms for measuring water quality and contamination events. For example, it may take at least 5 min to manually count a pour plate with more than 100 cfu, whereas the time to count the same plate using a digital colony counter is less than a minute including the time to place the plate on the counter and type in the sample details prior to data acquisition, Some data exists for HPC counting [9], [10], [16]; however, no data appear to be available for chromogenic agar-based technologies applied to water samples. One hundred routine samples were selected from a chlorinated filtered water distribution system over summer and winter (3 months) and samples recording a count of zero were eliminated from the data set. Typical positive recovery on MI agar was in the range of 1–80 cfu/100 mL, and HPC from 1 to 300 cfu/1 mL. Parallel replicate counting between experienced staff members (*n* = 15) familiar in the techniques and then again by the digital counter were undertaken to verify expected counting performance and reproducibility of both methods (Fig. 1, Fig. 2). This represented a total of 1500 counts for each method, which is recommended for testing repeatability and reproducibility [17], and signifies the variability of operators and digital counter (Table 1). Further repeatability of the samples (*n* = 25) was assessed using experienced senior staff (*n* = 6) independently measuring a marked rotated plate 10 times double blinded for each method (*n* = 1500, Table 2).

The resultant data showed no significant differences in the counting methods between digital and manual counting for measurement of water quality compliance for HPC and MI agar (Table 3, Table 4). The sensitivity and specificity of automated counting was comparable to manual counting (Table 5).

Reliable colour discrimination (*n* = 1036) using digital analysis linked to laboratory information management systems for chromogenic agars is required to avoid lengthy confirmation of isolates and would be a major advantage to the water industry worldwide.

Colony counting of HPC and MF techniques (chromogenic agars) can be considered critical analyses in terms of compliance and response to water quality incidents. Fast TAT, consistency and quality of the counts can be achieved using the latest digital analysis with confidence by water quality authorities.

## Conflict of interest

On behalf of the authors (we) certify that there is no conflict of interest with any financial organization regarding the material discussed in the manuscript. All ethical guideline responsibilities and the terms of this arrangement have been reviewed and approved by SA Water Corporation.

## Figures and Tables

**Table 1:** Comparison of manual and digital counts for HPC and MI methods – replication 100 samples counted by 15 staff members.

	*n*	Mean	95% CI	SE
**HPC**				
Operator	1500	335.180	269.250 to 401.110	33.6114
Digital counter	1500	363.377	289.104 to 437.650	37.8645
**MI**				
Operator	1500	16.4	15.9 to 16.9	0.25
Digital counter	1500	16.5	16.0 to 17.0	0.25

**Table 2:** Comparison of HPC and MI counts for manual against digital methods – repeatability 25 samples counted by 6 senior staff members and the plate rotated 10 times.

	*n*	Mean	95% CI	SE
**HPC**				
Operator	1500	1216.555	909.371 to 1523.739	155.9826
Digital counter	1500	1320.239	985.464 to 1655.015	169.9932
**MI**				
Operator	1500	20.478	19.941 to 21.015	0.2740
Digital counter	1500	20.460	19.924 to 20.996	0.2732

**Table 3:** Significance analysis of replication and repeatability (digital method vs. manual method) counting of HPC and MI chromogenic agar.

	HPC		MI	
	KW-ANOVA	rs	KW-ANOVA	rs
Replication (*n* = 1500)	>0.05	(0.92)	>0.05	(0.89)
Repeatability (*n* = 1500)	>0.05	(0.98)	>0.05	(0.95)

**Table 4:** Mean relative difference analysis (trial method vs. manual method) of paired sample results for water samples analyzed.

*n* = 1500	HPC	MI
Std dev	42.43	34.38
*U* (expanded uncertainty)	3.63	0.00
*x*_L_	−5.20	−2.53
*x*_H_	2.05	1.02
Outcome	Not different	Not different

**Table 5:** Specificity and sensitivity analysis (digital method vs. manual method) of HPC and MI chromogenic agar results.

*n* = 1500	HPC	95% CI	MI	95% CI
Sensitivity – TP	0.981	0.963 to 0.992	0.98	0.971 to 0.987
Specificity – TN	0.902	0.786 to 0.967	0.905	0.832 to 0.953
FP	0.098	0.033 to 0.214	0.095	0.047 to 0.168
FN	0.019	0.008 to 0.037	0.02	0.013 to 0.029

TP, true positive; TN, true negative; FP, false positive; FN, false negative.
